# Medical Student Experience and Outcomes, as Well as Preceptor Experience, with Rapid Conversion of a Preclinical Medical School Course to a Remote-Based Learning Format in the Setting of the COVID-19 Pandemic

**DOI:** 10.1007/s40670-021-01379-8

**Published:** 2021-09-03

**Authors:** David Grand, Victor L. Schuster, James M. Pullman, Ladan Golestaneh, Amanda C. Raff

**Affiliations:** 1 Albert Einstein College of Medicine, Bronx, NY USA; 2 Department of Medicine, Division of Nephrology, Albert Einstein College of Medicine/Montefiore Medical Center, Bronx, NY USA; 3 Department of Pathology, Division of Anatomic Pathology, Albert Einstein College of Medicine/Montefiore Medical Center, Bronx, NY USA; 4 Zucker School of Medicine - Northwell Lenox Hill Hospital, New York, NY USA

**Keywords:** Medical education, Pre-clinical, Remote learning, Renal, COVID-19 pandemic

## Abstract

**Objectives:**

To assess student outcomes and experiences, as well as preceptor experiences, after emergently converting a preclinical medical school renal course to a remote setting during the COVID-19 pandemic.

**Methods:**

First-year medical student examination scores and responses to Likert-scale questions on end-of-course evaluations from the 2018–2019 (traditional) and 2019–2020 (remote) academic years were compared. Free-text responses from students and preceptors were analyzed using a qualitative summative approach to extract major themes in perceptions of remote learning.

**Results:**

Mean student scores on course examinations did not significantly differ between the traditional and remote settings (*p* = 0.23 and 0.84 respectively). Quantitative analysis of student evaluations revealed no significant difference across all items in mean Likert-scale responses. Student and preceptor free-text responses identified course leader engagement and responsiveness as essential to the success of remote-based learning. Optimal group size and online etiquette are areas that require attention.

**Conclusions:**

Despite rapid conversion of a preclinical medical school renal course to a remote-based format in the setting of the COVID-19 pandemic, student scores and evaluations remain positive and largely unchanged.

## Introduction

The evolving COVID-19 pandemic disrupted many aspects of medical education and required physical distancing of the preclinical coursework which traditionally involves congregating students and course preceptors in close quarters. This pandemic forced many academic institutions to adapt classwork into a virtual setting. Clinical experiences and didactics which were expected to take place in the traditional face-to-face setting were emergently switched to an online setting whereby students and preceptors, separated by location, interact through electronic devices [1, 2].

Remote-based learning has been a subject of concern given difficulties in communication, easy distractibility, and technological difficulties. The abrupt conversion during the COVID-19 pandemic raised additional concerns, namely the ability to deliver high-quality education in a new remote setting with short notice; this encouraged some medical educators to call for the creation of pandemic response teams to minimize disruptions in medical education and ensure that educational objectives are met [3]. It has already been documented that student perceptions remained positive despite the rapid conversion from the traditional to remote classroom setting as a result of the COVID-19 pandemic [4]. What is lacking in the literature is an assessment of how student scores have been affected as a result of this rapid conversion.

The first-year preclinical renal course at Albert Einstein College of Medicine in the Bronx was emergently converted from the traditional setting to a remote e-course in a short span of time. The main objective of this study is to assess how student scores were impacted by the abrupt change in a preclinical course at a single institution. In addition, we assessed student and preceptor perceptions of this rapid transition with the goal of identifying best practices for the design and implementation of virtual medical school courses.

## Methods

### Study Design

For this descriptive fully inductive theory development study [5], available student data from identical multiple choice exams, student perceptions via course evaluation feedback, and faculty comments gathered via survey were analyzed.

### Course Reorganization

The renal organ system course at the Albert Einstein College of Medicine begins in early April and ends in early June. The course consists of 21 lectures, five large group sessions, and six small group sessions. In the traditional setting, lecturers teach in large auditoriums, and preceptors facilitate large and small group sessions face-to-face in either a large education center or smaller classrooms. Two exams and a low-stakes Team-Based Learning (TBL) quiz contribute to the final grade.

The COVID-19 pandemic necessitated conversion to remote learning in mid-March 2020, leaving just a few weeks to transition the renal system course into a remote setting. This was the first organ system course in the preclinical medical student curriculum to be converted online. Pre-recorded lectures from the previous academic year served as substitutions for live lectures, while large and small group sessions were converted to real-time Web-based Zoom experiences whereby students and faculty could attend from a remote location. Given the surge in the number of nephrology consultations and need for renal replacement therapy in New York City during the time of this course [6], nephrology clinical duties markedly increased. Hospital admissions in New York City in late March/early April 2020 peaked at over 1500 each day and deaths per day approached just over 600 [7]. This left fewer preceptors than usual to facilitate large and small group sessions. Daily Zoom-based office hours with either a senior faculty member or near-peer student tutor were added as novel features to the remote course. Identical multiple-choice question exams to the prior year were administered. Both classes took the computer-based examinations on the same exam platform but the 2018–2019 class was proctored in person while the 2019–2020 class was proctored through Zoom. The low-stakes TBL quiz component was not analyzed as it was not identical (Table 1).

**Table 1:** Comparison of 2018–2019 and 2019–2020 educational settings

	**2018**–**2019** **Traditional setting**	**2019–2020** **Remote setting**
Lecture setting	- Live auditorium	- Pre-recorded lectures from the prior year
Small group sessions	- Setting: small classrooms - Group size: 15 students per classroom - More preceptors available	- Setting: Zoom breakout rooms - Group size: 15 students per breakout room - Fewer preceptors available; rotating between breakout rooms
Large group sessions	- Setting: large education center - Group size: 6–7 students per table - More preceptors available	- Setting: Zoom breakout rooms - Group size: 6–7 students per breakout room - Fewer preceptors available; rotating between breakout rooms
Office hours	- In person - Appointments with faculty as needed	- Via Zoom - Daily office hours with faculty and near-peer tutor
Examination setting	- Auditorium setting - Proctored in person	- Remote setting - Proctored on Zoom

### Participant Recruitment

First-year MD students take both course examinations. MD/PhD students join the course only for the second half; thus, more students take exam 2 than exam 1 in any given year. The medical school annually collects student evaluations, including a series of 12 Likert scale and several free-text questions, that assess fulfillment of educational objectives, as well as strengths and areas of improvements for the course. Student and faculty evaluations were of particular interest to compare student perceptions between the two years as a guide to improve future remote course offerings. Evaluations were identical between the 2018–2019 and 2019–2020 academic years except for the addition of a free-text question on the experience with the remote nature of their learning in the 2019–2020 evaluation. Student grades and perception data were provided by deidentified educational records for the 2018–2019 and 2019–2020 academic years. In 2018–2019, all students answered Likert scale questions and had the option of answering all the free-text questions. Given concerns for evaluation burden, distribution of the course survey items was modified in the 2019–2020 academic year, such that all students had the option to respond to the free-text questions, but a statistically significant random sample comprising 35% of the class were provided the Likert scale questions.

Eligible preceptors were recruited to the study via an email which included a link to a voluntary Qualtrics survey. Consent was obtained prior to beginning the survey. Three faculty members (VS, JP, AR) and one student author (DG) were excluded from the survey. This anonymous survey consisted of a series of Likert scale and free-text questions that queried educational utility, positive and negative aspects, and overall experience with the virtual setting. Additionally, two senior faculty members (VS, JP) who led optional unstructured virtual office hours were interviewed for reflections on the virtual office hours. The Albert Einstein College of Medicine Institutional Review Board approved this research as an exempt study.

### Statistical Methods

#### Student Scores

Using Stata version 15.1, the mean score (percent) and standard deviation on each exam from years 2018–2019 and 2019–2020 were compared using Student’s *t*-test after assumptions for normal distribution of scores and equal variance between time periods were checked (Table 2).

**Table 2:** Comparison of 2018–2019 and 2019–2020 mean student test scores

**Exam**	**2018**–**2019**	**2019**–**2020**	***p*-value**
Exam 1, *N* = student number, mean (SD)	*N* = 166 82.2% (8.6%)	*N* = 169 81.1% (8.5%)	0.23
Exam 2, *N* = student number, mean (SD)	*N* = 179 80% (8.6%)	*N* = 184 79.8% (7.7%)	0.84

*SD* standard deviation

#### Student and Preceptor Evaluations

Likert-scale items for both student and preceptor evaluations ranged from 1 (unsatisfactory) to 4 (excellent). The weighted mean of scores for each student evaluation item was calculated for both the 2018–2019 and 2019–2020 academic years. Student’s *t*-test with unequal variance was used to compare the two years (Table 3) [8, 9]. Themes from both student and preceptor evaluations were extracted from free-text responses. Free-text responses were categorized using a summative approach by two authors (AR, DG) as belonging to one or more of the following themes which were established after an initial review of the range of comments:

- Clinical relevance: approaching course material using a clinical framework
- Course director/communication: management of the course by the course director
- Group active learning: learning content and experiences of small and large group sessions
- Lecture/course content: lecture and syllabus material, speakers, and practice material
- Organization: ordering of lecture and group sessions and layout of the online educational platform
- Media/technology: experiences with lecture recordings and Zoom

**Table 3:** Comparison of 2018–2019 and 2019–2020 student evaluations

**Survey item**	**2018–2019 (*N* = 176)**	**2019–2020 (*N* = 47)**	***p*-value**
Course learning objectives were clearly written	3.61	3.57	0.99
Quality of the course preparatory materials promoted learning	3.58	3.55	0.99
Course materials were well organized in curriculum management system	3.58	3.62	0.99
Course materials were available in a timely manner	3.64	3.55	0.98
Topics were organized in a logical sequence that facilitated learning	3.45	3.53	0.98
Clear learning objectives were explicitly provided for each instructional session	3.58	3.60	1.00
Lectures contributed to learning of course material	3.57	3.53	0.99
Small group ‘active learning’ sessions enabled me to apply course concepts	3.57	3.44	0.97
Large group ‘active learning’ sessions enabled me to apply course concepts	3.48	3.49	1.00
Course director regularly communicated with students and was responsive to student communications	3.63	3.59	0.99
Course content was made clinically relevant	3.70	3.64	0.99
Overall course was effective in advancing my learning	3.63	3.60	0.99

Representative quotes that best encompassed the themes listed above were selected by two authors (AR, DG). Student satisfaction with each theme was assessed by comparing the number of comments that considered the theme as a strength to the number of comments that suggested the theme needed improvement in the free-text responses.

## Results

A detailed comparison of the traditional and remote settings for the renal organ system course is shown in Table 1.

### Student Scores

Student exam scores are summarized in Table 2. There was no significant difference between mean examination scores in exam 1 and 2 (82.2% (SD 8.6%) vs 81.1% (SD 8.5%) and 80% (SD 8.6%) vs 79.8% (SD 7.7%); *p* = 0.23 and 0.84 respectively).

### Student Perceptions

Student perception Likert-scale responses extracted from course surveys of 176 out of 179 students (98.3% response rate) in the 2018–2019 academic year and 47 out of a 35% sample from the class size of 184 students (73.4% response rate) in the 2019–2020 academic year are summarized in Table 3. All comparisons of mean Likert scores showed no significant differences between the two academic years (all *p* > 0.97).

Optional free-text comments elicited from 176 out of 179 students (98.3% response rate) students in 2018–2019 and 146 out of 184 students (79.3% response rate) in 2019–2020 were compared. The ratio of number of comments on each course theme as a strength to that theme as an area of improvement is shown in Table 4.

**Table 4:** Comparison of 2018–2019 and 2019–2020 student evaluations

**Theme**	**Comment**					
	2018–2019			2019–2020		
	Strength	Improvement	Ratio*	Strength	Improvement	Ratio*
Clinical relevance	13	1	13:1	10	0	10:0
Course director/communication	5	4	1.5:1	20	0	20:0
Group active learning	20	4	5:1	32	10	3.2:1
Lecture/course content	12	25	0.48:1	14	27	0.52:1
Organization	34	11	3:1	42	10	4.2:1
Media/technology	0	2	0:2	0	0	0:0

^*^Ratio of number of comments on each course theme as a strength to that theme as an area of improvement

Student free-text responses on the unique virtual nature of the 2019–2020 course identified both strengths and weaknesses. Representative comments are presented in Table 5.

**Table 5:** 2019–2020 student free-text responses regarding virtual course organized by course theme

**Group active learning** *Student engagement* • “The groups that I enjoyed the most were ones where I had classmates that were talkative as it allowed us to talk through the cases more so than just go through the questions by typing a Google Doc. I think this was one downside to not being in person as it may be easier to kind of just sit there and type the answers without talking through it fully.” • “Overall, this course translated very well into a student-guided experience, and I personally preferred the Zoom large-group sessions over sessions from previous courses that were held in-person since there was increased student involvement and engagement.” *Online etiquette* • “I think it would be helpful to encourage/require students to turn on their videos during small groups. It was often frustrating to end up in small groups where only a couple of the students were participating because students realized they could get attendance credit by simply logging into zoom and using the word docs that other students worked on during the session.” • “There were many times when the facilitator was absent and we were left confused with whether we can leave or we should wait for a facilitator. It would have been very helpful to have facilitators check in more regularly with the students.” *Group size* • “Please make the small group session smaller, and add more preceptors. Groups of 12 + especially on zoom where the dynamic is different are hard to get everyone involved and stay focused on the task.”
**Lecture/course content** • “The video-guided sessions worked very well.” • “With no lecture time, a zoom review of the week’s lectures would be helpful, rather than question-based sessions.” • “Don’t use pre-recorded videos and charge the same tuition.”
**Media/technology** • “I have to give credit to the faculty and IT staff for making sure that our conferences all happened in such a well-organized way.” • “I found that there needs to be more instruction of professors on how to use zoom (especially recording software) to ensure timely use of the session….Sometimes there were tech issues, but overall we were pretty successful.”

### Preceptor Perceptions

Of the 13 eligible course preceptors, 12 completed the online survey. Based on the survey, 91% of the preceptors agreed or strongly agreed that they could provide high-quality teaching in Zoom-based settings, 91% agreed or strongly agreed Zoom-based sessions have educational utility in teaching students, and 90% agreed or strongly agreed that students were engaged in the Zoom-based settings. Representative preceptor free-text responses on their experiences with the 2019–2020 course and reflections of two senior faculty members excluded from the preceptor survey who participated in the virtual office hours are provided in Table 6.

**Table 6:** 2019–2020 preceptor reflections on virtual sessions and office hours

**Group active learning** *Student engagement* • “All students with video were engaged in learning activities. Some students had audio and video switched off during the whole session so it’s hard to estimate their involvement.” • “For the most part, many students were as engaged as in person.” *Online etiquette* • “Make everyone have [their] cameras on, I [think] some students have them off and they were not actually participating in the activity” • “Strongly encourage or require the students to show their faces on zoom” *Group size* • “To have 5–6 people in 1 zoom room is more effective than 12–15 people.” • Recommendation: “Small group < 5 students”
**Comparison to in-person teaching** • “It was more difficult than face to face teaching” • “The students are less interactive” • “Biggest difference was that I had to jump more between groups due to fewer preceptors.” • “Time wasted moving between [breakout] rooms.”
**Office hours** • “Office hours were a useful way to respond to student needs in two ways. They provided an opportunity to answer in as much detail as necessary questions from individual students on course material. They also allowed for additional review of material in the syllabus for the entire class, or as many as responded to the zoom invite for this purpose.” • “No students availed themselves of my office hours, however it was not uncommon for one or more students to ask to stay “live” after the [large or small group] session ended in order to clarify items or ask additional questions.”

## Discussion

Remote-based medical education is not a new concept. Interest in technology-based educational opportunities has grown rapidly in the last decade with many institutions recording and distributing video lectures to students to watch at the time, location, and rate of their convenience [10, 11]. As a component of medical curriculum reform and the release of new technology in recent years, medical institutions have implemented a wider array of remote-based learning modalities, including interactive online digital microscopy lessons [12] and Zoom-based small group clinical problem-solving sessions [13].

Despite a rapid and drastic change in course setting at our institution, average student exam scores remained largely unchanged. This is consistent with the results of a meta-analysis performed by the United States Department of Education demonstrating slightly better scores in remote-based learning when compared to in-person learning in both undergraduate and graduate courses [14]. This meta-analysis included a study on a medical histology course that demonstrated no significant differences in student scores between virtual and traditional microscopy cohorts [15]. Additionally, our results align with another study that reported no changes in average examination scores in a surgery clerkship following a transition to problem-based learning and didactics in the setting of the COVID-19 pandemic [16].

The data on student perceptions in the remote setting is mixed. At one institution, a switch to remote-based learning resulted in greater attendance, an increase in student voluntary contributions, higher quality of student discourse, and higher faculty satisfaction [13]. At another institution, 43.9% of students considered interaction with teachers during remote classes as poorer than in the physical classroom setting, and 50.9% considered physical classes as better than remote classes [17]. During the COVID-19 pandemic, 63.4% of students at one institution were satisfied with online teaching [18] and at another 68.6% of students noted a preference to continue > 90% of the learning online following the COVID-19 pandemic [19]. Our analysis concurs with students reporting overall satisfaction with remote learning while highlighting the need for course directors to remain engaged and responsive to students’ needs in remote educational settings. Advantages identified by students included a greater role for collaborative note-taking programs, the ability to connect students to a greater number of peers and preceptors than the traditional course via the Zoom breakout group randomization feature, and the unique advantages of using embedded video instruction as part of a live Zoom event. Other institutions have documented students’ preference for videocasted lectures uploaded in advance, providing them more flexibility in course preparation [20].

Medical educators have highlighted the importance of maintaining the collaborative nature of medical education in the remote setting, so it is essential to continue to utilize electronic resources that reproduce the advantages of classroom-based learning [21, 22]. This positive feedback influenced our medical institution’s approach to the 2020–2021 academic year with a traditional-remote hybrid approach, in which a majority of group-based learning took place via Zoom.

Despite these advantages, student feedback highlighted areas that require improvement. Students prefer live lectures as opposed to re-use of lectures recorded from prior years, or if not possible, some form of real-time lecture review. Additionally, there were concerns from both students and preceptors about online etiquette, most commonly citing the video-off feature as a disruption to the team dynamic of the small and large group sessions. Many believed that those who left their cameras off contributed minimally, if at all, to the sessions. These concerns about online etiquette clearly impacted both student and preceptor perceptions on student engagement and have been documented in other educational settings [23]. Though the number of students per group remained unchanged from prior years, both students and preceptors shared the opinion that larger Zoom breakout groups interfered with student engagement and educational utility. Both students and preceptors prefer smaller group sizes and consider an ideal group size as 5 to 6 students in the virtual realm. This is consistent with prior studies which demonstrated medical students prefer to work in smaller groups of 5 participants over larger groups of 15 [24]. Technological glitches and preceptor unfamiliarity with technology were additional concerns as has been documented in the literature, in which issues regarding technology remain a major source of student dissatisfaction with remote-based learning [12, 19]. Independent of unfamiliarity with technology, preceptors considered movement between breakout rooms as a hindrance to providing high-quality education.

Individual students did not often engage in the daily office hours offered. However, many students remained online following group sessions to ask questions and did attend office hours when an open invitation for a structured course review was offered as a response to a perceived need by many students for additional instruction. This highlights the importance of assessing student learning gaps, encouraging preceptors to remain available for questions following group sessions, and flexibility in providing avenues for one-to-one conversations with faculty in a remote setting. Virtual office hours and review sessions have been identified among the more favorable components of a remote curriculum [20].

This analysis has taught us many lessons about executing remote learning. It is best to continue to offer real-time lectures instead of re-using recorded ones. Preceptors should be trained to be familiar with the technology. Smaller remote group sizes than the traditional setting may be necessary. It is best to minimize the number of groups assigned to each preceptor; ideally, one preceptor should be assigned to one group. Lastly, ground rules for online etiquette must be established, including requirements for all members to keep their video cameras on during active learning sessions.

This study provides perspective on conversion of a medical course to a virtual setting in an emergency situation, including stable student satisfaction and exam score outcomes. Strengths of this study include data of exam scores and student surveys from the majority or statistically meaningful portion of the class, as well as a high response rate for the preceptor survey. Limitations include the inherent limitation of a report from a single course from a single institution and the lower overall response rate for the group of students in the COVID-19 affected academic cohort. Like many evaluations, the narrative comments are not mandatory so there could be selection bias in the opinions expressed. However, this may be balanced as it might be expected that students with both highly positive or negative comments are more likely to take time to reply.

## Conclusion

Despite rapid conversion of a preclinical medical school organ system course in the setting of the COVID-19 pandemic, student scores and evaluations remain positive and largely unchanged. This analysis is reassuring in that even in the midst of educational disruption, high-quality education that meets educational objectives can be achieved. In addition, it offers lessons in elements of remote learning that are applicable to future remote-based didactics, even in a non-emergency situation, including sustaining clear communication with students, keeping group sizes smaller for active learning sessions, maximizing the preceptor-to-student ratio, establishing clear online etiquette, and using live lectures in place of recycled ones. This analysis demonstrates that it is possible to fulfill course objectives by hosting a medical school course in a remote setting. Although certain aspects of remote-based learning require improvement in the future, remote-based learning shows promise as a means to substitute for traditional in-person learning when needed.

## Data Availability

Not applicable.
